# The Effect of SkitoSnack, an Artificial Blood Meal Replacement, on *Aedes aegypti* Life History Traits and Gut Microbiota

**DOI:** 10.1038/s41598-018-29415-5

**Published:** 2018-07-23

**Authors:** Kristina K. Gonzales, Stacy D. Rodriguez, Hae-Na Chung, Margaret Kowalski, Julia Vulcan, Emily L. Moore, Yiyi Li, Stephanie M. Willette, Yashoda Kandel, Wayne A. Van Voorhies, F. Omar Holguin, Kathryn A. Hanley, Immo A. Hansen

**Affiliations:** 1 Department of Biology, New Mexico State University, Las Cruces, NM 88003 USA; 2 Institute of Applied Biosciences, New Mexico State University, Las Cruces, NM USA; 3 Molecular Biology Program, New Mexico State University, Las Cruces, NM USA; 4 Department of Computer Science, New Mexico State University, Las Cruces, NM USA; 5 Department of Plant and Environmental Sciences, New Mexico State University, Las Cruces, NM USA

## Abstract

Public health research and vector control frequently require the rearing of large numbers of vector mosquitoes. All target vector mosquito species are anautogenous, meaning that females require vertebrate blood for egg production. Vertebrate blood, however, is costly, with a short shelf life. To overcome these constraints, we have developed SkitoSnack, an artificial blood meal replacement for the mosquito *Aedes aegypti*, the vector of dengue, Zika and chikungunya virus. SkitoSnack contains bovine serum albumin and hemoglobin as protein source as well as egg yolk and a bicarbonate buffer. SkitoSnack-raised females had comparable life history traits as blood-raised females. Mosquitoes reared from SkitoSnack-fed females had similar levels of infection and dissemination when orally challenged with dengue virus type 2 (DENV-2) and significantly lower infection with DENV-4. When SkitoSnack was used as a vehicle for DENV-2 delivery, blood-raised and SkitoSnack-raised females were equally susceptible. The midgut microbiota differed significantly between mosquitoes fed on SkitoSnack and mosquitoes fed on blood. By rearing 20 generations of *Aedes* exclusively on SkitoSnack, we have proven that this artificial diet can replace blood in mosquito mass rearing.

## Introduction

*Aedine* mosquito species transmit viruses which cause diseases such as yellow fever, dengue fever, chikungunya fever, and Zika congenital syndrome^1,2^. While the burden of mosquito-borne disease is centered in the tropical and subtropical parts of the world, recent range expansions of the principle disease vectors, *Aedes aegypti* and *Ae*. *albopictus*, have put the southern regions of North America and southern Europe at risk as well^1,3–10^. The lack of antiviral treatments or a licensed vaccine, for dengue, chikungunya and Zika virus means that vector control is the most effective strategy to safeguard populations against infection. However the efficacy of insecticide-based vector control is waning due to high levels of insecticide resistance in vector insect populations, lending further urgency to efforts to develop novel vector control strategies^11,12^. One promising approach, Sterile Insect Technique (SIT), has been successfully used in past insect pest eradication programs. The New World Screwworm (*Cochliomyia hominivorax*), the Mediterranean fruit fly (*Ceratitis capitate*), and the Tsetse fly (*Glossina morsitans*) have been successfully targeted with this technique^13–15^. Recent mosquito SIT approaches focus on genetically altering mosquito males for sterilization. In field trials RIDL (release of insects carrying a dominant lethal)-mosquitoes, have succeeded in reducing mosquito populations^16,17^. Progress in gene editing techniques, in particular CRISPR/Cas 9, has opened up new avenues for the usage of gene-drive systems in SIT^18–20^. Another promising approach to control mosquito-borne disease is the release of mosquitoes infected with the bacterial endosymbiont *Wolbachia*. *Wolbachia* has been shown to make mosquitoes more resistant to certain pathogens and to rapidly spread through populations due to a cytoplasmic incompatibility phenotype that confers a reproductive advantage to infected mosquitoes^21–25^.

All of the above-mentioned strategies require the mass-production of insects. Moreover, research into vector competence for vector-borne pathogens, such as recent efforts to determine the New World vectors for Zika virus, also requires substantial numbers of mosquitoes^26^. To mass produce mosquitoes, live vertebrates or collected vertebrate blood is currently necessary since all important vector species require vertebrate blood for reproduction^27,28^. Such blood can be offered to females through skin-feeding or through an artificial membrane feeding system, respectively^29^. Importantly, using vertebrate animals as blood source is subject to extensive regulations. Bioethics committees encourage the implementation of the 3Rs: replacement, reduction and refinement when it comes to animal use and recommend the use of artificial blood feeders instead of live animals^30^. Purchasing and storing blood for mass rearing programs is expensive and can be difficult under field conditions, particularly because blood has a two-week shelf life. Therefore, artificial blood replacements that can sustain a mosquito colony can be an attractive alternative to authentic vertebrate blood for rearing programs for blood-sucking insects^31^.

The challenge, however, is to develop an artificial meal that meets the following requirements as reviewed in Gonzales and Hansen (2016)^31^: First, mosquito females must imbibe the meal, meaning it must contain an adequate phagostimulant, and they must feed until satiated. Second, the meal must support oogenesis, demonstrated by the deposition of consistently large egg clutches. Next, the eggs generated from this meal should have a high viability and resulting adults should have have high fecundity. More generally, offspring resulting from a female fed on an artificial meal should be fit. Lastly, the offspring’s behavior or physiology should not be negatively altered^31,32^. When mosquitoes are to be used for studies of vector competence, the impact of rearing on the artificial diet on vector competence must be defined, and the vehicle should only be used for pathogen delivery to vectors if subsequent levels of infection are similar to those seen when the pathogen is delivered in blood.

We have used these requirements as a foundation for the formulation and testing of our artificial blood meal, SkitoSnack. Numerous attempts to develop an alternative blood meal substitute for mosquitoes have been reviewed in Gonzales and Hansen (2016)^31^. Recently, a red blood cell-based liquid artificial diet for *Wolbachia*-infected mosquitoes has been developed^33^. Previous studies found that serum proteins are sufficient for egg development, resulting in bovine serum albumin being the major protein source in most current diet formulations^32,34,35^, but red blood cells or another iron source are important for egg viability^32,33^.

Here, we report the development of SkitoSnack, an artificial diet produced in powdered form that, when rehydrated as a liquid meal, supports production of large batches of viable eggs in *Ae*. *aegypti* females. We also report that mosquitoes reared solely on SkitoSnack for ten generations or more exhibit similar life history traits as those raised on blood. We report differences in the midgut microbiota between blood-fed and SkitoSnack-fed mosquitoes and demonstrate that SkitoSnack is an appropriate vehicle for dengue virus delivery.

## Results

### SkitoSnack development strategy

We performed an extensive series of experiments to optimize the mixture of nutrients for SkitoSnack for *Ae*. *aegypti* females in order to maximize egg numbers and hatch rates. We also screened for a suitable phagostimulant, a buffer system, the best source of iron and the best nutrient source.

### Identification of a suitable phagostimulant

**Figure 1:**
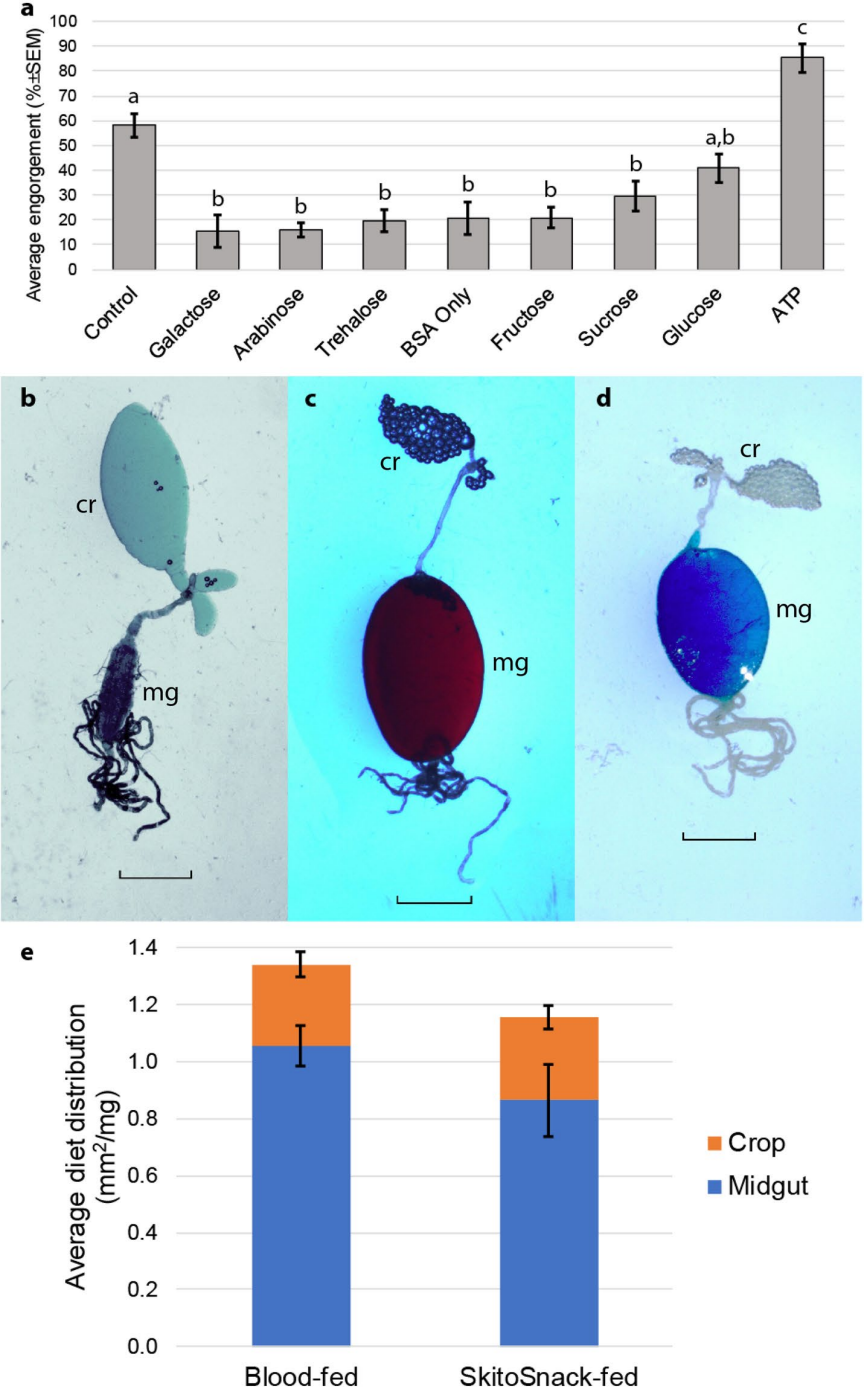
Effectiveness of phagostimulants and meal distribution within the mosquito alimentary canal. ta) Average percent engorgement rates of *Ae*. *aegypti* offered bovine blood (control), BSA only (in mAPS buffer and no phagostimulant), and BSA meals supplemented with galactose [50 mM], arabinose [50 mM], trehalose [50 mM], fructose [50 mM], sucrose [50 mM], glucose [50 mM], and ATP [3 mM]. Bars are arranged from weakest to strongest phagostimulant and represent average percent engorgement of at least 3 replicates ± SEM. Different letters indicate a statistical significant difference (p = 0.05 or less). (**b**–**d**) Alimentary canal showing crop (cr) and midgut (mg) of (**b**) females fed on sugar, (**c**) females fed on blood, and (**d**) females fed on SkitoSnack (blue hue is the result of the addition of food dye). Scale bar represents 1 mm. (**e**) Diet distribution of a bovine blood or SkitoSnack meal offered to 12^th^ generation blood-raised or SkitoSnack-raised females. To account for body scaling anomalies, the area of the midgut and crop were divided by individual body weight. Bars represent average area of midgut or crop (mm^2^) per body weight (mg) ± SEM of least 4 replicates.

An effective phagostimulant is critically important for artificial mosquito diets^31^. Previous artificial diets have used ATP^32,34,36^. ATP is relatively stable in frozen solutions but not at room and higher temperatures. Therefore, we designed a series of experiments to identify other, more stable phagostimulants. For this screen we added various chemicals to 200 mg/ml bovine serum albumin (BSA) solution and tested for phagostimulation using a feeding assay. We tested the sugars arabinose, fructose, galactose, glucose, sucrose and trehalose in three different concentrations (50 mM, 100 mm, and 1 M) and compared them to bovine blood (positive control), bovine serum albumin (BSA) alone (negative control), and BSA solution with 3 mM ATP. We found that none of the sugars was as strong a phagostimulant as ATP (Fig. 1a). Feeding rates on BSA solutions with 50 mM glucose concentration were not statistically significant different from those achieved with bovine blood. We therefore decided to use a combination of ATP and glucose in our final SkitoSnack recipe with the rationale that glucose will ensure high mosquito engorgement rates even when ATP is degraded over time.

### Identification of a suitable buffer system

In previous studies, a BSA meal dissolved in *Aedes* physiological saline (APS) buffer produced high mosquito engorgement rates^32,37^. We sought to alter the chemical components of APS to more closely mimic human blood and replace HEPES with sodium bicarbonate as the main buffering agent. We used feeding assays to determined engorgement rates of BSA/ATP solutions made with three different buffers: a simple bicarbonate (sodium bicarbonate [23 mM]), APS (sodium chloride [150 mM], sodium bicarbonate [0.1 mM], potassium chloride [4 mM], magnesium chloride [0.6 mM], calcium chloride [1.7 mM] and HEPES buffer [25 mM]), and a modified APS (mAPS) (sodium chloride [150 mM], sodium bicarbonate [23 mM], potassium chloride [4 mM], calcium chloride [2.5 mM], and magnesium chloride [0.8 mM]). While we found no significant difference in engorgement rates between the three buffers, a simple (One-way ANOVA, F(3,12), p = 0.002) and modified sodium bicarbonate APS buffer (mAPS) (One-way ANOVA, F (3,12) p = 0.025) produced high engorgement rates when compared to blood, and was selected because it provided more salts than the simple bicarbonate solution (Supplementary Fig. 1).

### Identification of a suitable iron source

Iron is important for egg viability in mosquitoes^31,38^. Therefore, we tested the effect of supplementing SkitoSnack with hemoglobin and more bioavailable forms of iron on egg viability. Oral iron supplements are more readily absorbed in humans when in the ferrous (iron (II)) oxidation state^39^. Therefore, we tested the effect of supplementing a basic BSA blood meal replacement with hemoglobin (Hb), iron (II) glutamate, iron (II) fumarate, and iron (II) sulfate on egg numbers and viability (Supplementary Fig. 2). While egg numbers were not affected by the different iron supplements, the egg viability increased 2-fold when hemoglobin was added to the meal.

### Identification of a suitable macronutrient sources

In an earlier study we found that blood serum proteins are critical part of the adult mosquito diet^32^ and other attempts to create artificial diets for mosquitoes have used BSA as the principal protein source^31^. In order to identify a potent mixture of macronutrients that supports large egg numbers and high egg viability, we supplemented our BSA/Hb solutions with chicken ovalbumin and chicken egg yolk in varying concentrations and combinations, fed them to female mosquitoes, and determined egg numbers and viability (Supplementary Table 1). The highest egg numbers and viabilities were recorded after feeding our BSA [200 mg/mL]/Hb [5 mg/mL] solution supplemented with both nutrients (chicken ovalbumin [50 mg/mL] and egg chicken yolk [5 mg/mL]), however, we chose to exclude ovalbumin from the final recipe because of its poor solubility in our mAPS buffer.

### SkitoSnack final recipe

The above results were used as the rationale to create the final SkitoSnack recipe. Table 1 shows the recipe that was used for all studies described below. From here on we will refer to our final recipe as ‘SkitoSnack’ and mosquitoes derived from eggs of females that have been fed on this diet as ‘SkitoSnack mosquitoes’. We determined the cost per milliliter of SkitoSnack using current product prices for each chemical component in the final recipe (Table 1). We found that all the components in SkitoSnack currently cost $0.22 per milliliter of liquid meal. An adult female mosquito can take up 2 to 4 times her own body weight in blood; some *Aedine* species have been reported to take between 2–18.6 µL of blood in a single feeding^40^. In our experience, *Aedes aegypti* takes usually between 1 and 2 ul of blood. Therefore, a milliliter of SkitoSnack can feed approximately 500 mosquitoes. Currently, the cost for purchasing defibrinated bovine blood is estimated between $0.11-0.60 per mL and $0.39-0.73 per mL for defibrinated rabbit blood (Hemostat Laboratories), however, this method requires blood to be repurchased every two weeks. Therefore, the cost of SkitoSnack falls into the price range of bovine blood and can be stored in its powdered form at −20 °C, indefinitely.

**Table 1:** Components, final concentrations, current cost per gram, cost per milliliter of SkitoSnack, company and product number of chemicals, and CAS number of all the items in our SkitoSnack recipe.

Components of SkitoSnack	Final Concentration	Final Concentration (g/mL)	Current cost per g ($)	Cost per mL of SkitoSnack ($)	Company-Product#	CAS #
Bovine serum albumin	200 mg/ml	0.2	0.892	0.178400	Reseach Products Intl.-A30075	9048–46–8
Bovine hemoglobin	5 mg/ml	0.005	0.525	0.002625	Sigma Aldrich-H3760	9008-02-0
Chicken yolk	5 mg/ml	0.005	0.478	0.002390	Sigma Aldrich-E0625	N/A
Glucose	50.0 mM	0.009008	0.019	0.000169	Sigma Aldrich-G7021	50-99-7
Adenosine triphosphate	3.0 mM	0.0016534	19.040	0.031481	Sigma Aldrich-A2383	34369-07-8
Sodium chloride	150.0 mM	0.008766	0.041	0.000359	Sigma Aldrich-S7653	7647-14-5
Sodium bicarbonate	23.0 mM	0.0019322	0.106	0.000205	Sigma Aldrich-S6297	144-55-8
Potassium chloride	4.0 mM	0.0002982	0.162	0.000048	Sigma Aldrich-P9333	7447-40-7
Calcium chloride	2.5 mM	0.0002774	0.104	0.000029	Sigma Aldrich-C1016	10043-52-4
Magnesium chloride	0.8 mM	0.0000761	0.053	0.000004	Sigma Aldrich-M8266	7786-30-3
TOTAL	1x	0.232		0.22		

### Shelf life of SkitoSnack powder

We performed a shelf life assay with SkitoSnack powder to determine how long powdered SkitoSnack could be stored and still produce engorgement rates comparable to blood when hydrated. We found that SkitoSnack can maintain engorgement rates comparable to blood for at least 84 days when prepared as a powder and stored at room temperature (25 °C) (Supplementary Fig. 3a**)**.

### Bench life of SkitoSnack solution

We performed a bench life assay to determine how long SkitoSnack could be used when hydrated and stored at 4 °C. We found that engorgement rates on a hydrated SkitoSnack meal were maintained for 3 hours after hydration when stored at 4 °C **(**Supplementary Fig. 3a). We observed a significant decrease in SkitoSnack engorgement at four (chi-squared, χ^2^ = 22.67, df = 1, p < 0.001) and five hours after rehydration (chi-squared, χ^2^ = 8.74, df = 1, p = 0.003). Therefore, we recommend feeding SkitoSnack solution within 3 hours after hydration.

### Diet distribution

We determined the distribution of different diets, blood, sugar solution, and SkitoSnack, within the alimentary canal of female mosquitoes. We found that sugar meals are directed to the crop while both, blood and SkitoSnack meals are directed to the midgut **(**Fig. 1b–d**)**.

### SkitoSnack supports egg production/viability comparable to whole blood

**Figure 2:**
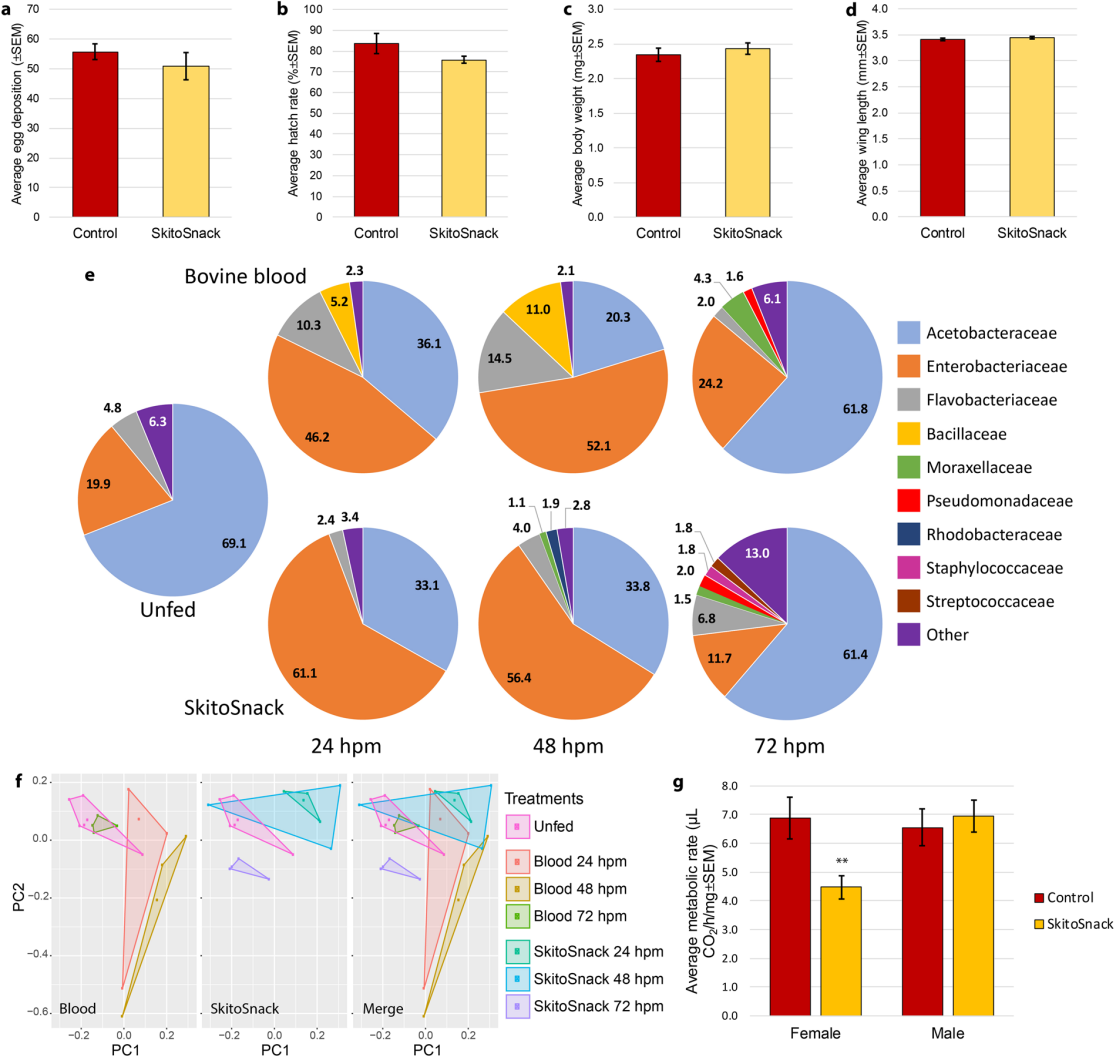
Life history traits and microbiota of SkitoSnack mosquitoes. (**a**) Average number of eggs laid from 11th generation bovine blood- and SkitoSnack-raised females. (**b**) Egg hatch rate comparison between 12^th^ generation bovine blood- and SkitoSnack-raised females. (**c**) The average weight comparison between 12^th^ generation bovine blood-raised and SkitoSnack-raised females. (**d**) The average wing length comparison between 12th generation bovine blood-raised and SkitoSnack-raised females. (**e**) Composition and percentage of bacterial communities in unfed and 24, 48 and 72 hours post meal (hpm) of bovine blood or SkitoSnack-fed female *Ae*. *aegypti*. The pie charts represent taxa at the family level. (**f**) Principle component analysis. Left panel: females that were unfed or offered a bovine blood meal, middle panel: females that were unfed (same group as in left panel) or offered a SkitoSnack meal, and right panel: merge. (**g**) Metabolic rate of females and males raised on blood or SkitoSnack. **Denotes statistical significance (P = 0.01).

To date, we have successfully raised a colony of *Ae*. *aegypti* (Ugal strain) for over 20 consecutive generations on SkitoSnack. A colony raised on bovine blood was reared in parallel and served as a control for all experiments. We fed SkitoSnack to 11^th^ generation *Ae*. *aegypti* SkitoSnack females (F11) and compared egg deposition numbers and hatch rates of their eggs (F12) with blood-fed control mosquitoes. The average numbers of eggs laid from females raised on both meals of the F11 generation were not significantly different (Fig. 2a) (t-test, t = 0.966, df = 21, p = 0.345). Egg hatch rates of the F12 generation were similar to hatch rates of blood-fed mosquitoes (Fig. 2b) (t-test, t = 1.534, df = 10, p = 0.156). The average wet body weight of F12 SkitoSnack females was not significantly different than their blood-raised counterparts (t-test, t = −0.825, df = 38, p = 0.415) (Fig. 2c) while their wing lengths were almost identical (t-test, t = −0.743, df = 38, p = 0.462) (Fig. 2d).

### SkitoSnack increases the diversity of bacterial communities in *Aedes aegypti* midguts

We analyzed the bacterial community of female midguts using 16S RNA analysis. We compared the midgut microbiota of unfed mosquitoes with those fed on bovine blood or SkitoSnack at 24, 48, and 72 hours after the meal **(**Fig. 2e**)**. We found that bacterial midgut communities change dramatically after both meals. *Enterobacteriaceae* relative abundance increases at 24 hours after ingestion of both meals, while *Acetobacteraceae* numbers decrease. We observed an expansion of *Bacillaceae* after blood feeding that did not occur within SkitoSnack-fed female midguts. We first tested patterns of diversity in the microbiota of mosquitoes subjected to different feeding regimes and found a significant interaction between meal (blood or SkitoSnack) and time post blood meal (pbm) on the diversity of bacterial communities, measured by the Shannon Wiener diversity index (H) (two-factor ANOVA, df = 2, p = 0.018). While the microbiota diversity in mosquitoes fed blood stayed fairly constant across time, the microbiota diversity in mosquitoes fed SkitoSnack peaked at 48 hrs pbm and declined sharply at 72 hrs pbm. We then analyzed the similarity in composition of the microbiota in different treatments. The microbiota of blood-fed mosquitoes was significantly different from the unfed cohort of mosquitoes at 24 (KS test, D = 0.076, p = 0.006) and 48 hours (KS test, D = 0.102, p = 6.3^−5^), but clusters with unfed mosquitoes at 72 hours (KS test, D = 0.030, p = 0.76) (Fig. 2f). The microbiota of SkitoSnack mosquitoes was found to be statistically significantly different from the unfed cohort at all time points (KS test: unfed vs. SS 24 h, p = 0.019; unfed vs. SS 48 h, p = 0.002; and unfed vs. SS 72 h, p = 8.12^−6^). The microbiotas of blood-fed and SkitoSnack-fed mosquitoes were similar at 24 (KS test, D = 0.030, p = 0.849) and 48 (KS test, D = 0.047, p = 0.323) hour time points but were significantly different at the 72-hour time point (KS test, D = 0.093, p = 0.001) (Fig. 2f).

### SkitoSnack females have a lower metabolic rate

We measured the metabolic rate of adult females and males raised on blood or SkitoSnack for 15 generations **(**Fig. 2g**)**. The feeding regime did not cause a significant difference in the metabolic rate of mosquito males, however SkitoSnack females had a significantly reduced metabolic rate.

### Eggs from SkitoSnack-fed females show differences in the metabolome compared with eggs from blood-fed females

Because bacterial symbionts are often important sources of vitamins and other metabolites in insects, we performed a study to compare the metabolomes of eggs from SkitoSnack-fed females with eggs from blood-fed ones. We compared the free metabolites of eggs from females raised on the two different meals for 14 generations **(**Supplemental Table 2**)**. There was a significantly higher amount of proline, tyramine, adenosine, arachidic acid, oleic acid, 5-β-cholestan-3-one, and zymosterol metabolites in the SkitoSnack-raised eggs than in the bovine blood-raised eggs. There was a statistically significantly *lower* amount of lactic acid in SkitoSnack-raised eggs.

### SkitoSnack females have the same DENV-2 vector competence as blood-raised females

We compared the dengue vector competence of females raised on blood with tenth generation SkitoSnack females to determine whether adult diet has an influence on the resulting offspring’s vector competence (Table 2). We found no significant difference between the body infection rates (Fisher’s exact test, df = 1, p = 1.0, N = 115), mean virus titer from infected bodies (t-test, t = −0.288, df = 17, p = 0.78), dissemination rates (Fisher’s exact test, df = 1, p = 0.58, N = 19), and mean virus titer from infected heads (t-test, t = 0.0126, df = 14, p = 0.99) of females raised on bovine blood or SkitoSnack challenged with a DENV-2-infected rabbit blood meal. There was a 16% and 17% infection rate for blood- and SkitoSnack-raised females, respectively and a 90% dissemination rate in the blood-fed cohort and a 78% dissemination rate in the SkitoSnack-fed cohort (Table 2: Experiment 1). We sought to replicate these findings by repeating the experiment using twelfth generation SkitoSnack females and found that it is repeatable. There was no significant difference in body infection rates (Fisher’s exact test, df = 1, p = 0.20, N = 40), mean virus titer from infected bodies (Mann-Whitney Rank Sum test, U statistic = 43.5, p = 0.71), dissemination rates (Fisher’s exact test, df = 1, p = 0.52, N = 21), and the mean virus titer from infected heads (t-test, t = 1.010, df = 16, p = 0.327) of females raised on blood or SkitoSnack challenged with a DENV-2-infected rabbit blood meal. There was a 39% and 64% infection rate for blood- and SkitoSnack-raised females, respectively, and a 100% dissemination rate in the blood-fed cohort and 79% dissemination rate in the SkitoSnack-fed cohort (Table 2: Experiment 2).

**Table 2:** The effect of rearing condition, blood meal vehicle, and dengue serotype on *Ae*. *aegypti* vector competence.

Experiment	Rearing Condition	Bloodmeal vehicle	Serotype-strain	N	% (No.) infected	Mean virus titer (±1SEM) from infected bodies (log_10_pfu/body)	% (No.) Disseminated (total)	Mean virus titer ( ± 1SEM) from infected heads (log_10_pfu/head)	% Infections leading to dissemination
1	Bovine blood	Rabbit blood	DENV-2 NGC Proto	62	16.1 (10)	3.3 ± 0.3	14.5 (9)	2.6 ± 0.3	90.0
	SkitoSnack	Rabbit blood	DENV-2 NGC Proto	53	17.0 (9)	3.4 ± 0.2	13.2 (7)	2.6 ± 0.4	77.8
2	Bovine blood	Rabbit blood	DENV-2 NGC Proto	18	38.9 (7)	3.6 ± 0.3	38.9 (7)	2.9 ± 0.3	100.0
	SkitoSnack	Rabbit blood	DENV-2 NGC Proto	22	63.6 (14)	3.3 ± 0.1	50.0 (11)	2.6 ± 0.2	78.6
	Bovine blood	SkitoSnack	DENV-2 NGC Proto	12	25.0 (3)	3.2 ± 0.2	25.0 (3)	2.5 ± 0.2	100.0
	SkitoSnack	SkitoSnack	DENV-2 NGC Proto	8	25.0 (2)	3.4 ± 0.05	25.0 (2)	2.5 ± 0.2	100.0
	Bovine blood	Rabbit blood	DENV-4 Dominica	9	66.7 (6)	2.7 ± 0.5	33.3 (3)	2.2 ± 0.3	50.0
	SkitoSnack	Rabbit blood	DENV-4 Dominica	20	20.0 (4)	2.6 ± 0.2	5.0 (1)	1.4	25.0

Table shows the rearing condition, bloodmeal vehicle, dengue virus serotype, sample size, percent infected, mean virus titer of infected bodies, percent dissemination, mean virus titer of infected heads, and percent infections leading to dissemination data of 10^th^ (experiment 1) or 12^th^ generation (experiment 2) bovine blood-raised or SkitoSnack-raised *Ae*. *aegypti* females offered a dengue 2 virus-infected blood meal in rabbit blood or SkitoSnack and a dengue 4 virus-infected blood meal in rabbit blood.

### SkitoSnack females have a lower DENV-4 vector competence than blood-raised females

DENV comprises genetically and phenotypically diverse constellation of lineages^41^, and it was therefore important to test whether the DENV-2 results could be replicated using a different dengue virus serotype. We challenged twelfth generation SkitoSnack females with DENV-4 in rabbit blood. We found a significant difference in body infection rates between blood- and SkitoSnack-raised females (Fisher’s exact test, df = 1, p = 0.03, N = 29). The blood-raised cohort had a body infection rate of 67%, whereas, the SkitoSnack-raised cohort was 20%. A nominal logistic regression using data from experiment 2 revealed a significant interaction between rearing condition (BB or SS) and virus serotype (DENV-2 or DENV-4) (DF = 1, Chisquare = 8.29, P = 0.004) on percentage of mosquitoes infected, indicating that the effect of being reared on SkitoSnack on vector competence depends on virus strain.

Despite the infection rate, there was no difference in the mean virus titer from infected bodies (t-test, t = 0.283, df = 8, p = 0.78). Interestingly, there was no significant difference in dissemination rates (Fisher’s exact test, df = 1, p = 0.57, N = 10). There was a 50% dissemination rate in the blood-fed cohort and a 25% dissemination rate in the SkitoSnack-fed cohort (Table 2: Experiment 2).

### SkitoSnack is an appropriate vehicle for dengue virus delivery

To compare vehicle conditions, we challenged twelfth generation blood and SkitoSnack females with DENV-2 in SkitoSnack or rabbit blood (Table 2: Experiment 2). The blood-raised cohort had an infection rate of 38.9% when DENV-2 was offered in rabbit blood and 25% when DENV-2 was offered in SkitoSnack, but in both cases the dissemination rate was 100%. The SkitoSnack-raised cohort had an infection rate of 63.6% and dissemination rate of 78.6% when DENV-2 was offered in rabbit blood, and an infection rate of 25% and dissemination rate of 100% when DENV-2 was offered in SkitoSnack. We found no difference in body infection rates, mean virus titer from infected bodies, dissemination rates, or mean virus titer from infected heads when blood- or SkitoSnack-raised mosquitoes were challenged with DENV-2 in rabbit blood or SkitoSnack (Table 2: Experiment 2).

## Discussion

Here we present an artificial blood-meal replacement suitable for the mass culture of *Aedes aegypti*. SkitoSnack, is chemically-defined and can replace vertebrate blood. It is an ethical alternative to the use of live vertebrate animals, is cost-effective, it supports high egg numbers and viability and most importantly, the resulting adults have similar life history traits as blood-fed mosquitoes.

We optimized the SkitoSnack recipe in a rational series of experiments. We first focused on finding novel phagostimulants because the commonly used ATP is relatively unstable. In these screens, we focused on naturally occurring sugars, because mosquitoes of both sexes acquire sugar from plant sources for energy throughout their adult life^42,43^. Glucose was the most effective of the sugars we tested so we added 50 mM glucose to our final recipe in addition to ATP to ensure a longer shelf life of SkitoSnack. Our second focus was to identify a suitable buffer system for SkitoSnack. In order for an artificial meal to be similar to whole blood it must have a balance of dissolved mineral salts and a buffer that is resistant to pH changes. Human blood possesses a bicarbonate buffering system^36,44^. We chose mAPS as the buffer for SkitoSnack because the potassium, bicarbonate, magnesium, and calcium concentrations of mAPS are within normal human adult serum levels^45^. mAPS does not contain the buffering agent HEPES, which was present in the original APS buffer^37^. We then chose to supplement SkitoSnack with commercially available bovine hemoglobin [5 mg/ml] as an iron source because the majority of iron absorbed by the mosquito is obtained from heme in blood^38^ and a One-way ANOVA test on the data for iron sources showed that *all* the iron sources were significantly different from blood (p < 0.001), but not significantly different from each other. However, we chose to supplement our meal with hemoglobin because its use resulted in the highest number of viable eggs in our screens in comparison to the other iron sources. Although, we do not have experimental evidence that the increase in egg viability is due to the iron or protein content of hemoglobin, we suspect that both are responsible. Next, we compared the efficacy of various mixtures of potential macronutrient sources. After screening two different candidates we identified that supplementation with at least one, or a combination of nutrients, resulted in better egg numbers and viability than BSA alone. However, the BSA/hemoglobin combination and chicken egg yolk produced the best egg numbers and viability when fed to mosquito females. Egg yolk contains high levels of cholesterol and other nutrients^46^ and is commercially available as a dry powder and cholesterol-supplementation has been found to be critically important for mosquito oogenesis^47^.

The final recipe for SkitoSnack is a mixture of ten components that are all commercially available. SkitoSnack is a brown powder that can be stored at room temperature and is mixed with distilled water before feeding. We recommend feeding the solution within three hours after preparation. The price of SkitoSnack is within the price range of comparable bovine blood from a commercial source (Hemostat Laboratories).

Mosquitoes take up sugary solutions in their crop, while blood is routed to the midgut^42,48^. We found that even though SkitoSnack has a high level of glucose, it is routed to the mosquito midgut and not the crop which we think is important for successful initiation of vitellogenesis and egg development^28^.

We have maintained an *Ae*. *aegypti* colony fed exclusively on SkitoSnack for over 20 generations. Life history traits including egg numbers, hatch rates, wet weight, and wing length of these SkitoSnack mosquitoes are very similar to the bovine blood-fed control colony. While BSA-based diets that enabled female mosquitoes to produce large egg batches have been described before, the viability of eggs derived from artificial meals has often been a problem because such eggs had severely reduced hatch rates^31,32,34^. In contrast SkitoSnack-fed mosquitoes produced eggs with equal viability to eggs derived from blood-fed mosquitoes.

Bacterial symbionts and commensals play important roles in mosquito digestion, nutrient uptake, reproduction, growth, development, immunity, and vector competence^23,49–51^. Mosquitoes obtain their gut microbiota from their environment during their larval stages and are ‘gut sterilized’ during metamorphosis and acquire a new microbiota after eclosion^52,53^. Adult blood feeding has been shown to reduce the abundance of certain microbes that have been acquired transstadially from the larval stages^54^. We found that the mosquito gut microbiota differs depending on diet. There was an increase in the relative abundance of *Bacillaceae* and *Flavobacteriaceae* bacteria after a blood feeding that did not occur when mosquitoes were fed on SkitoSnack. At the completion of the gonotropic cycle, 72 hours after feeding, when the meals are completely digested and all leftovers excreted, the microbiota of the SkitoSnack-fed females showed a greater diversity of bacterial families present than in the blood-fed females. Interestingly, the genus *Asaia* comprises a range of 96.1–99.7% of the *Acetobacteraceae* family represented in all time points. Previous research has shown that the presence of bacteria in the *Asaia* genus are mutually exclusive with *Wolbachia*^55^ and may inhibit the maternal transmission of *Wolbachia* in *Anopheles* mosquitoes^56^. In our study, the abundance of *Asaia* is found in comparable amounts in the control (blood) and SkitoSnack cohorts. We do not suspect that SkitoSnack will influence the maternal transmission of *Wolbachia*, but future testing with *Wolbachia*-infected *Aedes* and other mosquito species is necessary to confirm this hypothesis.

Successful SIT strategies depend on the ability of sterilized males or *Wolbachia*-treated females to disperse and mate with wild mosquitoes in the natural population^57,58^. We found that the female mosquitoes raised from eggs of SkitoSnack-fed females had a significantly lower metabolic rate than females raised from eggs of blood-fed females. This may have a negative impact on *Wolbachia*-related SIT strategies because it has been shown that the metabolic rate of female *Culex tarsalis* after blood feeding is tied to reproductive fitness (i.e. enzyme secretion, vitellogenesis initiation, and excretion of by-products)^59^. Therefore, further alterations to the SkitoSnack recipe, and additional testing with *Wolbachia*-infected mosquito colonies, is necessary. In contrast, we found that the metabolic rates of male mosquitoes raised from eggs of SkitoSnack-fed females was not significantly different from mosquitoes raised from eggs of blood-fed females. This suggest that SkitoSnack does not adversely affect the metabolism of male offspring and male mosquitos which is important for male-focused mosquito SIT strategies.

The aim of the metabolomics study was to determine whether eggs raised on SkitoSnack lacked metabolites important for egg viability. Both groups of metabolomes were remarkably similar with only seven out of 400 metabolites assayed showing significantly different concentrations. Two of the metabolites up-regulated in SkitoSnack-derived eggs, 5-β-cholestan-3-one and zymosterol are part of the steroid biosynthesis pathway^60,61^. We hypothesize that this is due to the incorporation of cholesterol-rich chicken egg yolk [5 mg/mL] in the final SkitoSnack recipe. Lactic acid is known as a byproduct of anaerobic respiration and mosquito attractant^62^ and the only metabolite that was down regulated in SkitoSnack-raised eggs. Lactic acid is also a human blood metabolite with normal concentrations ranging around 2 mM/L^63^. Further research is needed to determine if adding lactic acid to SkitoSnack affects lactic acid levels in mosquito eggs and if it has an effect on egg viability.

A critical question when considering using artificial blood-meal replacements to sustain laboratory strains is if females raised on it have changed susceptibility to pathogens^64,65^. We found that rearing mosquitoes on SkitoSnack or delivering virus in a DENV-2 infected SkitoSnack meal did not affect infection or dissemination rates. Interestingly, when females that were raised on blood or SkitoSnack were challenged with a DENV-4-infected rabbit blood meal, there was a significantly lower infection rate in SkitoSnack-raised females. It has been shown that large-bodied adult *Ae*. *albopictus* females may show less susceptibility to DENV-2 than smaller ones^66^. However, we found no significant difference in the size of our SkitoSnack females in comparison to blood raised females. Our finding suggests that dengue virus serotype influences *Ae*. *aegypti* vector competence. We found that SkitoSnack could be an appropriate vehicle for DENV-2 infection because there was no significant difference in infection and dissemination rates and the mean body and head virus titers of females, raised on blood or SkitoSnack, challenged with DENV-2 delivered in SkitoSnack. Interestingly, a recent study found that Zika virus infection of *Ae*. *aegypti* was significantly lower when mosquitoes were offered a Zika virus isolate in an artificial blood-meal replacement^67^. Further testing with other dengue serotypes and pathogens is necessary to gain a complete picture of the interplay between artificial blood meal replacements and vector competence in mosquitoes.

In summary, with SkitoSnack we have developed an artificial blood-meal replacement that can be prepared as a powder and stored at room temperature with a long-shelf life and used to rear large numbers of *Ae*. *aegypti* without compromising egg numbers, egg viability, and the fitness of offspring. It is important to note that we used a well-established laboratory strain of *Ae*. *aegypti* in our studies. Additional studies are necessary to confirm that SkitoSnack is also effective in other laboratory strains and field-collected populations. This work lays the foundation for the development of artificial blood-meal replacements for other mosquito species and other blood-sucking arthropods. Further testing of life history parameters such as mosquito longevity, flight mill tests, mating success, male mating competitiveness, and egg storage time should be conducted before implementation of SkitoSnack in vector control mass rearing facilities.

## Methods

### Mosquito rearing

Mosquito rearing was carried out according to Gonzales *et al*. 2015^32^. Briefly, *Ae*. *aegypti* (UGAL) eggs were vacuum-hatched and first instar larvae were transferred to pans containing distilled water. Larvae were maintained on pellets of Special Kitty cat food (Wal-Mart Stores, Bentonville, AR). Pupae were transferred into 30 cm^3^ cages (BugDorm, Taiwan) with 50 ml Erlenmeyer flasks containing 10% sucrose solution with a cotton wick. At three days post eclosion mosquitoes were fed on defibrinated bovine blood (Hemostat, Dixon, CA) using an artificial blood feeding system (see below). Three days after feeding eggs were collected by introducing a half-full plastic water cup lined with germination paper. The paper containing the eggs was removed from the cage, air dried and stored at least one week before hatching. Insectary conditions were maintained at 27.5 °C, 16 L: 8D cycle, and 70% relative humidity (RH).

### Mosquito feeding

At 7–10 days post eclosion approximately 25 female mosquitoes were transferred from the stock rearing 30 cm^3^ cage into 15 cm^3^ cages (BugDorm, Taiwan) and starved for at least 16 hours. The mosquitoes were fed defibrinated bovine blood (Hemostat Laboratories, CA) or artificial diet, SkitoSnack, via water-jacketed feeders (Prism Research Glass Inc., Raleigh, NC). The feeders were attached to a 37 °C circulating heated water bath (Lab Companion, Biotechnical Services, Inc.). A parafilm membrane (Parafilm “M” laboratory film, WI) was stretched over the bottom open end of each glass feeder and 3 mL of each meal was pipetted into the top narrow opening. Each meal reached 37 °C before the glass feeders were placed on top of each mosquito cage. Feeding of each meal lasted for 30 minutes.

### Individual Mosquito egg collection

Engorged females were selected and individually placed in 50 ml conical Falcon™ centrifuge tubes that contained a damp piece of mosquito germination paper on top of a water soaked cotton ball, hereafter termed egg chambers. The females remained in the egg chambers for 5 days. On the 6^th^ day, the females were killed and the germination paper or filter paper containing the eggs were removed and placed in petri dishes and stored in an insect environment chamber for 7 days to desiccate. During the 7 days of desiccation, the eggs were counted manually with the use of a light dissection microscope and a handheld counter.

### Artificial diets

SkitoSnack chemical components were diluted in ddH_2_O in 15 mL round-bottom tubes. Tubes were vortexed to mix components and placed in 37 °C water bath and vortexed occasionally to yield a brown-colored aqueous solution. All chemicals were purchased from Sigma-Aldrich. Feedings were carried out as previously described.

#### Phagostimulant Study

Naturally occurring carbohydrates arabinose, fructose, galactose, glucose, sucrose, and trehalose were tested. Each phagostimulant tested was dissolved in a 200 mg/mL BSA meal prepared in a simple bicarbonate buffer (sodium bicarbonate [23 mM]) on the day of feeding to produce 50 mM, 100 mM, and 1 M final concentrations. Additionally, the phagostimulatory effects of BSA alone (no phagostimulant added) and ATP was used at 3 mM as Galun (1967) suggests that ATP concentrations between 1 mM and 10 mM produce high engorgement rates^36^.

#### Macronutrient Study

Each nutrient tested was added to our SkitoSnack base formula (BSA 200 mg/mL, Glucose [50 mM], dissolved in mAPS buffer). Bovine hemoglobin [5 mg/mL], chicken ovalbumin [50 mg/mL], and chicken egg yolk [5 mg/mL] were prepared on the day of feeding and were tested alone and in combination. All chemicals were purchased from Sigma-Aldrich, St. Louis, MO. Feedings were carried out as previously described.

### Maintenance of mosquito colonies

Two groups of approximately 30, 16-hour starved, female mosquitoes were separated from the bovine blood-fed culture 30 cm^3^ cage and transferred to a 15 cm^3^ mosquito cage. To initiate and maintain the colony, one group was fed whole bovine blood, and served as the control, and the other group was fed SkitoSnack through an artificial membrane feeding system (described above) for 1 hour. After feeding, a damp filter paper placed on top of water-soaked cotton balls inside a petri dish was placed inside the cage for egg deposition. The filter paper was collected 5 days after feeding and desiccated for 7 days inside an insect environment chamber. After desiccation, the eggs were vacuumed hatched in 100 ml of distilled water in a 1000 ml Erlenmeyer flask. This process was carried out for 20 consecutive generations.

### Diet distribution measurements

Adult female mosquitoes raised on SkitoSnack for 12 generations were fed a blood or SkitoSnack meal through an artificial membrane feeding system for 30 minutes. Immediately after feeding, fully engorged females were anesthetized on ice and the crop and midgut of each female were dissected in *Aedes* physiological saline with forceps under a dissecting microscope. Each intact crop and midgut was measured using an Olympus SZX12 stereo microscope and Infinity Analyze and Capture software (Version 6.2, Lumenera Corp.). Before each measurement, the software was calibrated using a micrometer scale (Ted Pella, Inc.). A picture of the crop and midgut sample was taken and the outline of the organs was drawn using the polygon tool. The Infinity software package was used to calculate the area and perimeter of each sample. Only intact crop and midgut sacks were measured.

### Mosquito life history trait measurements

#### Wing length and body weight

1^st^ and 12^th^ generation eggs of bovine blood-raised and SkitoSnack-raised mosquitoes were vacuumed hatched for 15 minutes in 100 ml of distilled water. The emerged larvae were transferred into a 45 cm × 30 cm × 6 cm shallow pan with 2 liters of water and given access to five pellets of cat food. The next day, the larvae were separated into four 45 cm × 30 cm × 6 cm shallow pan each containing 500 larvae and each pan was given five pellets of cat food. The four pans were monitored daily and cat food was added as needed. The pupae were transferred into a 11.5 cm diameter × 3.5 cm deep round plastic cup, filled with distilled water, and placed inside of a 30 cm^3^ mosquito cage for emergence. 7–10 day old females were starved for 16 hours and two random samples of 20 females were taken from each cage, one sample of mosquitoes was weighed individually and the wing lengths of the other sample was measured individually.

#### Egg numbers

To determine egg numbers, a group of approximately 30 females were separated from the 1^st^ or 11^th^ generation cage and were offered their respective meals. Each fully engorged female was placed, alone, inside an egg deposition chamber for five days. On the sixth day, the females were killed with CO_2_ and individual egg papers were collected and the deposited eggs were counted with a handheld counter under a light microscope.

#### Hatch rate

10 females from the 1^st^ or 12^th^ generation cage was separated into a 15 cm^3^ mosquito cage and offered their respective meals, bovine blood or SkitoSnack, for 20 minutes through an artificial membrane feeding system. After feeding, the females were given access to a 20% sucrose-soaked cotton ball and a large egg deposition tray so they could lay *en masse*. Egg papers were removed from cages on the sixth day and desiccated for 7 days. 100 plump eggs from each egg paper was collected with a paintbrush under a light microscope and counted with a handheld counter. The eggs were vacuumed hatched in 100 mL of distilled water in a 1 L Erlenmeyer flask connected to a filtration vacuum pump (KNF Laboport® mini-pump, Sigma Aldrich).

### SkitoSnack powder shelf life assay

Thirty-three grams of a powdered SkitoSnack formula (BSA, hemoglobin, chicken yolk, glucose and ATP) were ground together with a mortar and pestle and collected in a 50-mL centrifuge tube. Modified *Aedes* physiological saline (mAPS) buffer was made as a liquid stock solution. Powdered SkitoSnack formula and mAPS buffer were stored at room temperature (25 °C). 2.1 grams of stock powdered SkitoSnack was dissolved in mAPS and mixing was carried out by vortexing and placing in 37 °C water bath for a total of 10–15 minutes. One drop of green food color (Market Pantry) was added to increase visibility through mosquito abdomen to a final volume of 9.5 mL of meal. 7–10-day old mosquitoes (Ugal strain) were starved for at least 16 hours prior to experiment. Approximately 20–30 females were placed into small cages (15 × 15 × 15 cm) before feedings were carried out. 3 mL of SkitoSnack and 3 mL of bovine blood were pipetted into separate glass feeders and fed via an artificial membrane feeding system by placing the feeders on top of a 15 cm^3^ cage for 1 hour. After feeding, the females were collected by a battery-powered insect aspirator and anesthetized on ice. Fully and partially engorged females were sorted and counted. Engorgement rates were calculated by dividing the number of fully plus partially engorged females by the total number of females in the cage. Feedings were carried out on days 0, 1, 2, 7, 14, 21, 31, 44, 48, and 84. Experiments were carried out on different days using different batches of laboratory stock mosquitoes.

### SkitoSnack working stock bench life assay

Mosquitoes and SkitoSnack solution were prepared as described above. Feeding assays were carried out at 0, 1, 2, 3, 4, and 5 hours after mixing. Experiments were carried out on different days using different batches of laboratory stock mosquitoes. The dissolved SkitoSnack solution was stored at 4 °C for the duration of the experiment. After feeding, the females were collected and analyzed as described above. After each feeding time point the glass feeders were rinsed with distilled water and the parafilm was replaced.

### Viruses and cell lines

C6/36 *Aedes albopictus* cells were maintained at 32 °C in minimum essential medium (MEM) supplemented with 10% heat-inactivated fetal bovine serum (FBS), L-glutamine [2 mM], nonessential amino acids [2 mM], and gentamyacin [50 µg/mL]. 1 mL of DENV-2 NGC Proto (8.17 log_10_pfu/mL) and DENV-4 D16 Dominica (8.66 log_10_pfu/mL) freshly thawed working stocks, raised on C6/36 cell monolayers as previously described^68^, were added to 14 mL (Experiment 1) or 2 mL (Experiment 2) of washed red blood cells, prepared from fresh, defibrinated rabbit blood (Hemostat Laboratories) supplemented with 10% sucrose or freshly prepared SkitoSnack meal and offered to females via an artificial membrane feeding system as previously described^68^.

### Mosquitoes, mosquito infection, and dissection

Groups of 10^th^ generation bovine blood-raised and SkitoSnack-raised females or 12^th^ generation bovine blood-raised and SkitoSnack-raised females were starved for 24 hours and offered a dengue virus- containing (DENV-2 NGC or DENV-4) blood meal for 20 minutes via an artificial membrane feeding system as previously described^68^. Samples of the infectious blood meal were collected before and immediately after feeding to quantify post-blood meal viral titer. The pre- and post-bloodmeal virus titer was 5.6 log_10_pfu/mL and 5.1 log_10_pfu/mL when DENV-2 was offered in rabbit blood and 6.7 log_10_pfu/mL and 6.1 log_10_pfu/mL, respectively, when DENV-4 was offered in rabbit blood. The pre- and post-bloodmeal virus titer was 6.5 log_10_pfu/mL and 5.6 log_10_pfu/mL, respectively, when DENV-2 was offered in SkitoSnack. After feeding, all females were chilled on a cold tray and fully engorged females were separated from unfed females and incubated at 27 °C and 80% RH on a 12:12 hour light:dark cycle for 14 days with *ad libitum* access to 10% sucrose. At the end of the incubation period, mosquitoes were cold killed and the head of each female was separated from the body with forceps under a dissecting light microscope, placed in a separate microcentrifuge tube, and homogenized with a motorized grinder and a plastic disposable 1.5 mL pestle in 250 µL of mosquito dilution media (100 mL Hanks balanced salt solution, 10 mL fetal bovine serum, 2 mL amphotericin B [250 µg/mL], 400 µL Ciprofloxacin [1% solution], and 50 µL Chleomycin [600 mg/4 mL solution]). All samples were stored at −80 °C before and after homogenization.

### Quantification of viral titer

As previously described^68^, confluent monolayers of C6/36 cells in 24-well plates were inoculated with serial 10-fold dilutions of homogenized mosquito body or head samples in cell culture medium and incubated at 32 °C for 2 hours. After incubation, 1 mL of optimethylcellulose (in OptiMem media supplemented with 2% fetal bovine serum, 2 mM of L-glutamine, and 0.05 mg/mL of gentamycin) overlay was added to each well and monolayers were incubated for 5 days at 32 °C. Viral plaques were visualized by immunostaining with DENV-2 or DENV-4 specific antibody.

### Mosquito midgut DNA extraction and purification

A cage of stock (bovine blood-raised) colony female mosquitoes were starved for 16 hours and separated into small 15 cm^3^ mosquito cage containing about 20 mosquitoes each. One cage of mosquitoes served as the control (“unfed bovine blood control”) and the others were fed their respective meals, bovine blood or SkitoSnack and dissected at the 24, 48 and 72-hour mark. The control group had six replicates and the bovine blood and SkitoSnack treatments each had four replicates for each time point. After feeding, mosquitoes were collected using a battery-powered aspirator and anesthetized on ice. The abdomen of five females were removed with dissection scissors and placed together into 1.7 mL microcentrifuge tubes containing 180 µL of tissue lysis buffer. The samples were homogenized using a motorized homogenizer and disposable pestle. Then 20 µL of proteinase K was added and incubated at 56 °C for at least 1 hour. Total DNA was extracted and purified using the DNeasy Blood and Tissue Kit (Qiagen) protocol. DNA was eluted with 50 µL of millipore water instead of the DNeasy Blood and Tissue Kit elution buffer. DNA concentrations were measured using a NanoDrop spectrophotometer and NanoDrop computer software (Thermo Fisher Scientific).

### Bacterial 16S rRNA PCR

The polymerase chain reaction (PCR) primers 515/806 were used to amplify 16 S ribosomal RNA gene V4 variable region. A 30 cycle PCR reaction using the HotStarTaq Plus Master Mix Kit (Qiagen, USA). The PCR parameters were 94 °C for 3 minutes, followed by 28 cycles (5 cycle used on PCR products) of 94 °C for 30 seconds, 53 °C for 40 seconds and 72 °C for 1 minute, after which a final elongation step at 72 °C for 5 minutes. Sequencing was performed by Mr. DNA (Shallowater, TX, USA) on an Illumina Hiseq platform. Data was processed using a proprietary analysis pipeline. Briefly, sequence barcodes, primers, short sequences (<150 bp), and ambiguous base calls and with homopolymer runs (exceeding 6 bp) were removed. Operational taxonomic units (OTUs) were inspected and aligned at 97% similarity. The final OTUs with 97% similarity were taxonomically classified using BLASTn against a curated database derived from GreenGenes, RDPII (http://rdp.cme.msu.edu) and NCBI (www.ncbi.nlm.nih.gov) and compiled into each taxonomic level into both “counts” and “percentage” files. Count files contain the actual number of sequences and percentage files contain relative percentages of sequences within each sample that map to the designated taxonomic classification.

### Mosquito metabolic rate measurement

Resting metabolic rates were measured on 7–10 day old mosquitoes. Mosquitoes were reared on either bovine blood or SkitoSnack for 14 generations. For metabolic measurements the mosquitoes were collected by aspiration from the mosquito cage, immobilized on ice, and divided by sex. The mosquitoes were allowed to recover from this cooling and were then re-immobilized with a stream of humidified nitrogen gas. An individual mosquito was then placed into a 2 ml glass measurement chamber with tweezers and the chamber was closed with a custom machined metal plug sealed with double O-rings. This process took less than a minute. The mosquitoes quickly recovered and began moving within a few minutes of immobilization. The chambers were then flushed for 30 seconds at a flow of 150 ml/min STPD with CO_2_ -free, 80% RH (at 27 °C) room air and left sealed for approximately 1 hour. At the end of the sampling period the chamber was flushed into a Li-Cor 6262 carbon dioxide gas analyzer (Li-Cor, Lincoln, NE). A total of 5 measurements were recorded for each mosquito. For the first 2 measurements, the chambers were filled with 80% RH CO_2_ free air. To determine the metabolic response of the mosquito to desiccating conditions the chamber was flushed with 0% RH air for the final 3 metabolic measurements. The amount of CO_2_ produced by each fly was calculated using DATACAN software (Sable Systems International, Henderson, NV). The five metabolic measurements could be highly variable. For this analysis, the lowest reading of the 5 measurements was used in the metabolic comparisons. A total of 16 mosquitoes from each sex and feeding regime were measured. Each set of experiments also included two chambers without mosquitoes to control for background CO_2_ production, or the presence of CO_2_ in the air used to flush the chambers. The CO_2_ concentration in these blanks was typically ≪5% of that measured in a chamber with a mosquito. The CO_2_ gas analysis system was zeroed daily against CO_2_ free air, and calibrated weekly against a 988 ppm certified gas standard (Air Products, Long Beach, CA.). The metabolic rates of the mosquitoes were measured on 2 consecutive days. After the different measurements, the mosquitoes were frozen at −80 °C in 2 ml vials sealed with a rubber stopper until they were weighed. All mosquitoes were weighed within 2 days of being frozen.

### Mosquito egg metabolomics

Total metabolites were extracted and analyzed as described by Fiehn and Lee with few modifications^69^. Briefly, approximately 5–8 mg of 14^th^ generation eggs were weighed and collected into 1.7 mL microcentrifuge tubes. Approximately 0.5 mL of sterile glass beads and 0.5 mL chloroform:methanol:water (10:3:1 v/v/v) were added to each mosquito egg sample and placed into a Precellys 24 homogenizer (Bertin Instruments, US) for 15 seconds. The samples, and beads, were centrifuged and the supernatant was collected into a separate 1.7 mL microcentrifuge tube. The supernatant was collected three times. The supernatant was then dried using a vacuum centrifuge (Eppendorf, US) for 2 hours. The next day, 3 µL of 20 mg mL^−1^ ribitol in pyridine, 5 µL of 1 mg mL^−1^ C4-C24 Even Carbon Saturated FAMEs (Sigma-Aldrich, US), and 45 µL of 20 mg mL^−1^ methoxyamine HCl:pyridine was added to the dried samples and placed in an incubating shaker for 1.5 hours at 37 °C. Then, 45 µL of MSTFA was added to each sample and placed in incubating shaker for another 0.5 hours at 37 °C. The samples were then centrifuged, and supernatant was transferred to 1.5 mL amber glass vials (Agilent, US) for chromatographic separation and mass analysis. Metabolites were separated with a 7890 A Gas Chromatography (GC) system (Agilent Technologies, US), and analyzed with a Leco Pegasus High Throughput time-of-flight mass spectrometer (TOF-MS) (Leco, US). Total ion chromatograms were deconvoluted with ChromaTof 4.41 (Leco, US), and metabolites were searched against the Fiehn library and metabolites were identified based on retention time (min) and mass-to-charge ratio (m/z). Unique ions were selected by a signal-to-noise (S/N) threshold of 50, and quantified by peak area. Spectral data were then aligned by the internal standard (ribitol) in MET-IDEA V2.08 (Noble Foundation, US). Peak area values were normalized to sample weight, and then log_2_ transformed for computational analysis. Data were uploaded to MetaboAnalyst 3.0 for both statistical analysis and metabolic pathway analysis^70^. The statistical analysis included t-tests to determine statistical difference (p < 0.05) in individual metabolites between SS and BB. Metabolites were examined in the context of biological pathways via pathway enrichment analysis, and identified significant fold change (p < 0.05) in SS vs. BB. Additionally, pathway topology analysis was performed to assess pathways in the context of structure and metabolic relevance.

### Statistical analysis

Phagostimulant, diet distribution, egg numbers, hatch rate, body weight, wing length, shelf life, and working stock data were tested for normality using a Shapiro-Wilk test. Phagostimulant data was analyzed by a one-way ANOVA, then a Holm-Sidak all pairwise multiple comparisons post test was conducted. Diet distribution, egg numbers, hatch rates, body weight, and wing length were analyzed using a T-test performed on SigmaPlot 12 statistical software (Systat Software Inc.). Bench life and shelf life data were analyzed using a Pearson’s Chi-squared test. Body infection and dissemination rate data was analyzed using the Fisher exact probability test performed on JMP Pro statistical software (SAS Institute Inc.) and Vassar stats online program (vassarstats.net). Body and head titer data was first evaluated for normality, and analyzed using a T-test if the data was normal, or a Mann-Whitney Rank Sum test if the data was not normal. Body and head titer data were analyzed using SigmaPlot 12 statistical software (Systat Software Inc.). All tests were performed with an overall significance level of α = 0.05. To analyze the microbial dataset, principle component analysis (PCA) was constructed using the R package ggfortify. PCA is a multivariate statistical technique that analyses inter-correlated observations that are transformed into orthogonal variables called principle components^71^. For this data, each point represents a column, which is the value of one repeated among all bacteria phylum. The Shannon-Weiner diversity index was calculated for each individual mosquito microbiota and the impact of feeding condition and time post blood meal (pbm) on diversity was tested via a 2 factor ANOVA implemented in JMP Pro statistical software (SAS Institute, Inc.). Microbiota similarities among treatments were analyzed using the Kolmogorov Smirnov (KS) Goodness-of-fit test by R function ks.test. Metabolic rates were analyzed using a multivariate analysis of variance (MANOVA) test with weight as a covariate. Egg metabolomic data was analyzed using MetaboAnalyst V3.0. The Log2 transformed data was uploaded for the basic set of univariate analyses under the “Statistical Analysis” tab. All statistically significant metabolite data were analyzed using a T-test (default), except for tyramine which was calculated using the Mann Whitney Rank Sum test.

## Electronic supplementary material

Supplemental Information
