# Supplementary material for: The Effect of SkitoSnack, an Artificial Blood Meal Replacement, on Aedes aegypti Life History Traits and Gut Microbiota
Source: Sci Rep. 2018 Jul 23;8:11023. doi: 10.1038/s41598-018-29415-5 (PMC6056539; doi:10.1038/s41598-018-29415-5)
Supplement: Supplementary file 1 — Supplemental Information [file 41598_2018_29415_MOESM1_ESM.pdf]

SCIENTIFIC REPORTS

The Effects of SkitoSnack, an Artificial Blood Meal Replacement for *Aedes aegypti*, on Mosquito Life History Traits and Microbiome

Supplemental Information

Kristina K. Gonzales[1], Stacy D. Rodriguez[1], Hae-Na Chung[1], Margaret Kowalski[1], Julia Vulcan[1], Emily L. Moore[1], Yiyi Li[4], Stephanie Willette[5], Yashoda Kandel[1], Wayne Van Voorhies[3], Omar Holguin[5], Kathryn A. Hanley[1], and Immo A. Hansen [1,2,3,*]

1 Department of Biology, New Mexico State University, Las Cruces, NM 88003

2 Institute of Applied Biosciences, New Mexico State University, Las Cruces, NM

3 Molecular Biology Program, New Mexico State University, Las Cruces, NM

4 Department of Computer Science, New Mexico State University, Las Cruces, NM

5 Department of Plant and Environmental Sciences, New Mexico State University, Las Cruces, NM

*corresponding author

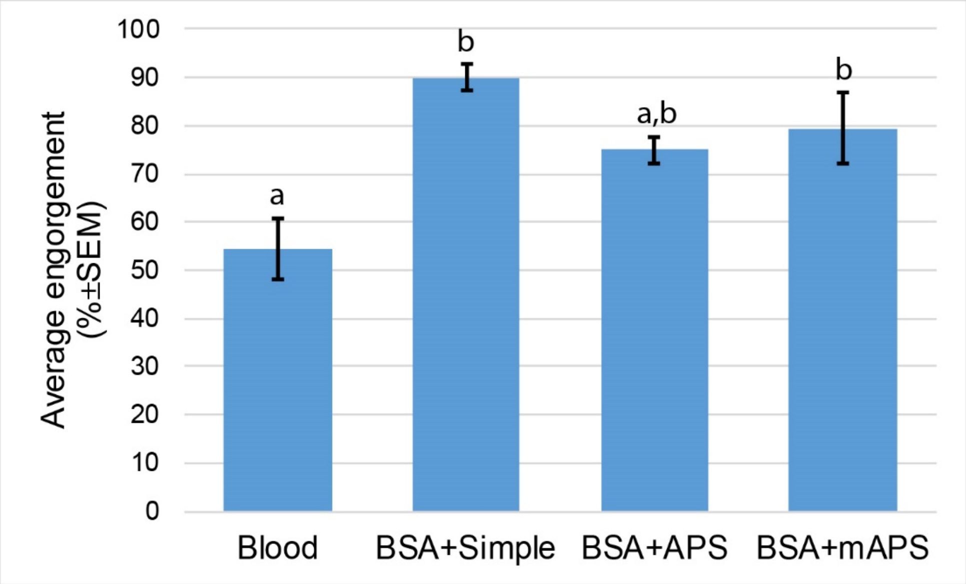

Supplemental Figure 1. **The effect of buffers on mosquito engorgement rates.** Percent engorgement of *Ae. aegypti* females on a BSA [200 mg/ml] meal dissolved in a simple bicarbonate buffer (sodium bicarbonate [23 mM]), *Aedes* physiological saline (APS) (sodium chloride [150 mM], sodium bicarbonate [0.1 mM], potassium chloride [4 mM], magnesium chloride [0.6 mM], calcium chloride [1.7 mM] and HEPES buffer [25 mM]), or modified *Aedes* physiological saline (mAPS) (sodium chloride [150 mM], sodium bicarbonate [23 mM], potassium chloride [4 mM], calcium chloride [2.5 mM], and magnesium chloride [0.8 mM]). Bars represent average engorgement of 4 replicates and error bars represent ±standard error of means (SEM). Different letters denote statistical significant difference ($p<0.05$).

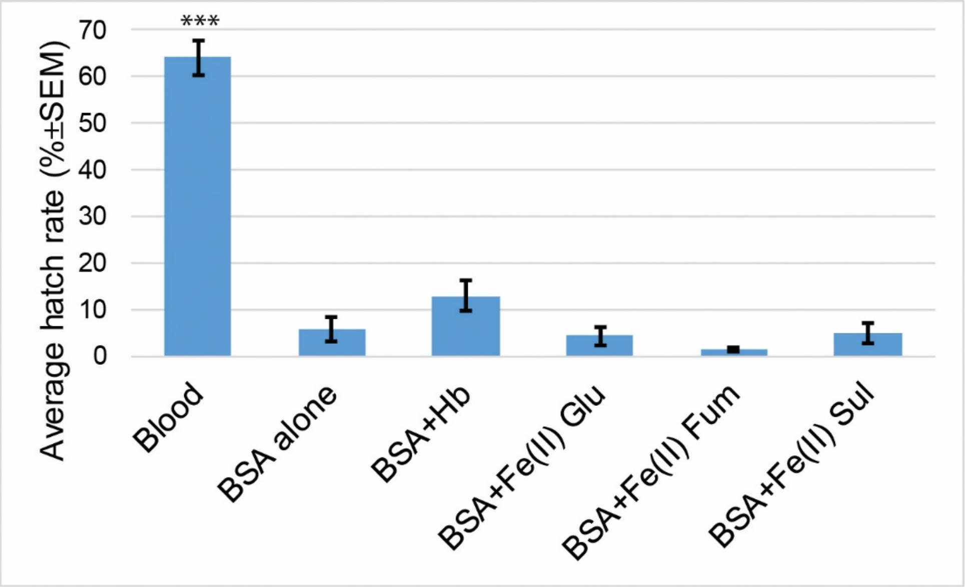

Supplemental Figure 2. **The effect of various iron supplements on mosquito egg hatch rates.** Average hatch rate of *Ae. aegypti* females given a blood, BSA alone [200 mg/mL], and BSA supplemented with 77.6 µM of various iron sources: bovine hemoglobin (Hb), Iron (II) gluconate (Fe(II) Glu), Iron (II) fumarate (Fe(II) Fum), and Iron (II) sulfate (Fe(II) Sul). This concentration was was chosen because it represents the molarity of hemoglobin at 5 mg/mL. Bars represent the average percent hatch rate of 3 replicates and the error bars represent ±standard error of means (SEM). ***denotes a statistical difference of $p<0.001$.

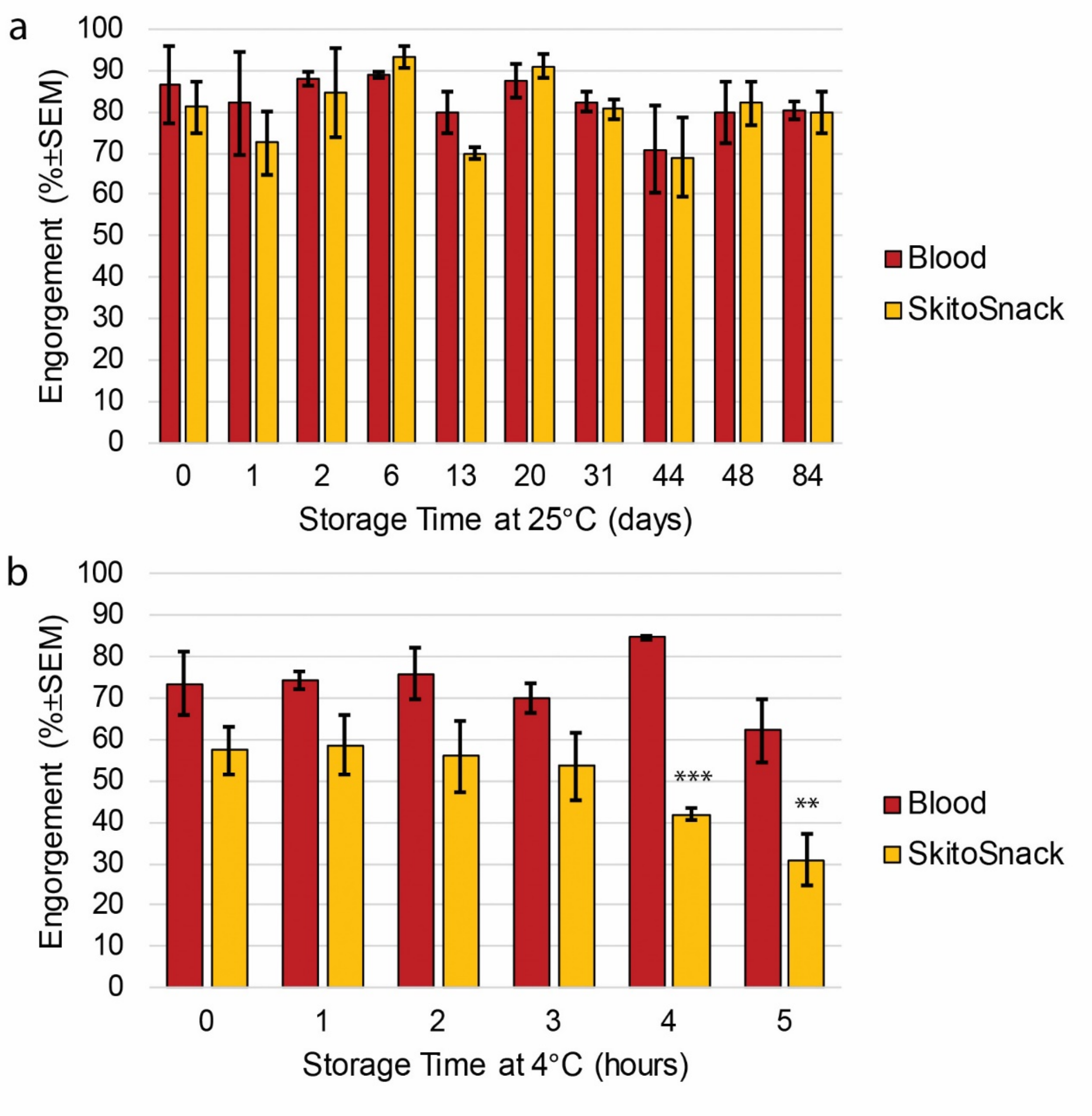

Supplemental Figure 3. **Shelf life and bench life of SkitoSnack.** a) Average engorgement rates of *Ae. aegypti* on a hydrated SkitoSnack stored at 25°C as a powder for various days and offered during the first hour of reconstitution. b) Average engorgement rates of *Ae. aegypti* on a hydrated SkitoSnack stored at 4°C during the first 5 hours of reconstitution. ***denotes a statistical difference of $p<0.001$ and **denotes a statistical difference of $p<0.01$. Both, shelf life and bench life data was analyzed using a Pearson's chi-squared test with $\alpha=0.05$.

	N	Total # Eggs Laid	Mean # Eggs/Female	# Larvae (7 dpm)	% Hatch
WB Bovine	13	909	69.9	650	71.5
BSA Alone	8	438	54.8	77	17.6
BSA+OA+CY	16	771	48.2	238	30.9
BSA+OA+Hb	6	377	62.8	68	18.0
BSA+OA+CY+Hb	12	653	54.4	362	55.4
BSA+CY+Hb	10	474	47.4	248	52.3

Supplemental Table 1. Fecundity and viability of female *Ae. aegypti* given various meals. Meals were supplemented with bovine blood, BSA [200 mg/mL] alone, and BSA supplemented with various combinations of macronutrients: chicken ovalbumin (OV) [50 mg/mL], chicken yolk (CY) [5 mg/mL], and bovine hemoglobin (Hb) [5 mg/mL]. Table shows total number of females that took a meal, total number of eggs laid, mean number of eggs laid per female, number of larvae present at 7 days post meal (dpm), and percent hatch rates. Table represents data from a single replicate.

Metabolite	P-value	Average BB (±SEM)	Average SS (±SEM)	Average BB Heat Map	Average SS Heat Map
proline	0.01**	16.9±0.2	18.0±0.2		
tyramine	0.06*	14.9±0.1	15.9±0.8		
adenosine	0.04**	15.1±0.1	15.8±0.2		
arachidic acid	0.03**	14.9±0.1	15.5±0.2		
oleic acid	0.10*	16.8±0.3	18.0±0.5		
lactic acid	0.10*	19.5±0.6	17.9±0.6		
5-β-cholestan-3-one	0.08*	16.1±0.3	18.0±0.9		
zymosterol	0.07*	16.0±0.2	18.4±0.9		

Heat Map Legend
**Minimum**
**Median**
**Maximum**

Supplemental Table 2. **Metabolites in mosquito eggs.** Data was acquired from mosquito eggs collected from mosquitoes fed bovine blood (BB) or artificial meal, SkitoSnack (SS). Metabolites were compared for the two separate treatments. Raw spectra were searched against the Fiehn library, and data were aligned to the internal standard (ribitol). Aligned values were $\log_2$ transformed, and then averaged for biological replicates (n=4). Standard error of the mean (SEM) is provided for averaged values, and a heat map was generated to compare average values. The heat map was generated based on minimum (red), median (black) and maximum (green) values (14.73, 16.87, 22.94, respectively). Metabolites were sorted based on functional groups into compound classes. T-tests were performed in MetaboAnalyst 3.0 to compare BB and SS samples, and p-values are provided. * denotes values of statistical significance $p \leq 0.10$ and ** denotes values of statistical significance $p \leq 0.05$.
